# Toxic Shock Syndrome Toxin 1 Induces Immune Response via the Activation of NLRP3 Inflammasome

**DOI:** 10.3390/toxins13010068

**Published:** 2021-01-18

**Authors:** Lianci Peng, Jiali Jiang, Tingting Chen, Dongyi Xu, Fengqing Hou, Qingyuan Huang, Yuanyi Peng, Chao Ye, Dong-Liang Hu, Rendong Fang

**Affiliations:** 1 Joint International Research Laboratory of Animal Health and Animal Food Safety, College of Veterinary Medicine, Southwest University, Chongqing 400715, China; penglianci@swu.edu.cn (L.P.); jiangjiali202012@163.com (J.J.); cttctt@outlook.com (T.C.); xudongyi29@gmail.com (D.X.); houfengqing08@163.com (F.H.); huangqingyuan2020@163.com (Q.H.); pyy2002@sina.com (Y.P.); yechao123@swu.edu.cn (C.Y.); 2 Chongqing Animal Disease Prevention and Control Center, Chongqing 401120, China; 3 Department of Zoonoses, Kitasato University School of Veterinary Medicine, Towada 034-8628, Japan; 4 Immunology Research Center, Medical Research Institute, Southwest University, Chongqing 402460, China

**Keywords:** *Staphylococcal* toxins, toxic shock syndrome toxin 1, inflammasome, interleukin-1β

## Abstract

*Staphylococcus aureus* is a Gram-positive opportunistic pathogen which causes infections in a variety of vertebrates. Virulence factors are the main pathogenesis of *S. aureus* as a pathogen, which induce the host’s innate and adaptive immune responses. Toxic shock syndrome toxin 1 (TSST-1) is one of the most important virulence factors of *S. aureus*. However, the role of nucleotide-binding oligomerization domain-like receptor family pyrin domain containing 3 (NLRP3) in TSST-1-induced innate immune response is still unclear. Here, purified recombinant TSST-1 (rTSST-1) was prepared and used to stimulate mouse peritoneal macrophages. The results showed that under the action of adenosine-triphosphate (ATP), rTSST-1 significantly induced interleukin-1β (IL-1β) and tumor necrosis factor-α (TNF-α) production in mouse macrophages and the production was dose-dependent. In addition, rTSST-1+ATP-stimulated cytokine production in macrophage depends on the activation of toll like receptor 4 (TLR4), but not TLR2 on the cells. Furthermore, the macrophages of NLRP3^−/−^ mice stimulated with rTSST-1+ATP showed significantly low levels of IL-1β production compared to that of wild-type mice. These results demonstrated that TSST-1 can induce the expression of inflammatory cytokines in macrophages via the activation of the TLR4 and NLRP3 signaling pathways. Our study provides new information about the mechanism of the TSST-1-inducing host’s innate immune responses.

## 1. Introduction

*Staphylococcus aureus* (*S. aureus*) is a common Gram-positive extracellular pathogen causing severe infections, such as abscesses, osteomyelitis, septic arthritis, pneumonia and endocarditis [1]. In the last few years, *S. aureus* pathogenesis to the host has been identified by different virulence factors, such as surface proteins (protein A), pore-forming toxins (e.g., Panton-Valentine Leukocidin (PVL)), α-hemolysin (α-toxin) and superantigen toxins [2,3,4]. During *S. aureus* infection, these virulence factors can induce innate immune responses and produce inflammatory or pro-inflammatory cytokines. However, among these virulence factors, the interaction between *Staphylococcal* superantigen toxins (SAgs) and host innate immunity is less studied.

Superantigens are the products of some bacterial exotoxins or retroviruses with a variety of immune activities [5]. Toxic shock syndrome toxin 1 (TSST-1) is one of SAgs [6]. TSST-1 is an important virulence factor of *S. aureus* and causes toxic shock syndrome (TSS) [7]. This disease can be diagnosed by fever, rash and desquamate, hypotension and organ impairment, leading to the high mortality [8]. TSST-1 directly crosslinks the major histocompatibility complex class II (MHC-II) molecules on antigen-presenting cells to T cell receptors (TCRs), resulting in the activation of T cells and macrophages with high production of cytokines [9,10]. Some studies showed that TSST-1 can enhance the lipopolysaccharide (LPS)-induced production of gamma interferon (IFN-γ), IL-1β, IL-6, IL-10 in human peripheral blood monocytes and rabbit spleen cells [11,12]. TSST-1 also increases LPS-induced IL-1 secretion in macrophages and TNF-α and IL-12 secretion in dendritic cells [13,14,15]. However, the mechanism of TSST-1-induced these inflammatory cytokines in the host cells is still unclear.

Inflammasome is a multiprotein complex that consists of apoptosis-associated speck-like protein containing a caspase-activating and recruitment domain (ASC), Nod-like receptors (NLRs) and pro-caspase-1. So far, inflammasome family members contain NLRP1, NLRP3, NLRC4, NLRP6, NLRP7, NLRP12 and absent in melanoma 2 (AIM2) [16]. These inflammasomes can be activated by different microbial stimuli to release inflammatory cytokines IL-1β and IL-18, which play an important role in the host’s innate immunity against microbial infection [17]. For example, NLRC4 is assembled in response to the flagellin of *Salmonella typhimurium* and *Legionella pneumophila*, while AIM is the receptor of intracellular bacterial DNA such as *Listeria monocytogenes* [18,19,20]. Among these inflammasomes, NLRP3 is one of the most extensively studied inflammasomes. The NLRP3 pathway can be activated by different bacteria, such as *Streptococcus pneumoniae*, *Pasteurella multocida*, *Corynebacterium pseudotuberculosis* and *S. aureus* etc. [21,22,23,24].

The classic activation pathway of NLRP3 needs two signals. The first signal is mainly triggered by bacteria or pathogenic molecules such as lipoprotein and LPS, which activate the Nuclear Factor kappa-light-chain-enhancer of the activated B cell (NF-κB) signaling pathway through TLR2 or TLR4, leading to production of pro-IL-1β and pro-IL-18, and the transcriptional and post-translational modification of NLRP3 [25]. A second activation signal, such as a bacterial toxin or adenosine-triphosphate (ATP), activates NLRP3 by K^+^ outflow and then causes the recruitment of ASC, followed the activation of caspase-1 [26,27,28]. Activated caspase-1 cleaves off pro-IL-1β and pro-IL-18, allowing them to be processed into mature IL-1β and IL-18, eventually resulting in their secretion to the extracellular environment [29]. It has been reported that α-hemolysin and the PVL of *S. aureus* toxins induced IL-1β via the activation of NLRP3 [30,31]. However, it is not clear whether TSST-1 can also activate the NLRP3 inflammasome like *S. aureus* other toxins.

In this study, we expressed and purified TSST-1, then investigated the role of TSST-1 in activation of the NLRP3 inflammasome in mouse primary macrophages. Under the action of ATP, mouse peritoneal macrophages were activated by TSST-1 via the activation of NLRP3, causing the maturation and release of IL-1β. Our study provides a better understanding on the interaction of TSST-1 and the host immunity, which contributes to the development of therapeutics against *S. aureus* infection.

## 2. Results

### 2.1. Expression and Purification of Recombinant TSST-1

**Figure 1:**
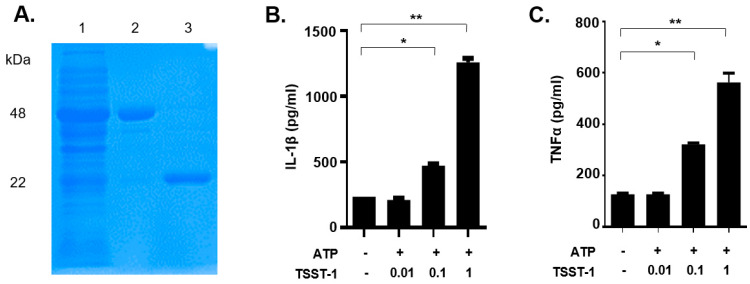
Purified rTSST-1-induced IL-1β and TNF-α expression in macrophages under the action of ATP. (**A**) The recombinant protein GST-TSST-1 was purified by Glutathione SepharoseTM 4B and digested by PreScission Protease. Lane (1): supernatant after the cleavage; lane (2): GST-TSST-1; lane (3): purified rTSST-1. Different concentrations of the purified rTSST were used to stimulate mouse macrophages under the action of 2 mM ATP. After stimulation, cell supernatants were collected and cytokines IL-1β (**B**) and TNF-α (**C**) were determined by ELISA. The data are represented as mean ± SD. of three independent experiments for each group (*n* = 3). * *p* < 0.05; ** *p* < 0.01.

Recombinant TSST-1 had been demonstrated to show similar activity to natural toxins purified from *S. aureus* [1]. In this study, recombinant TSST-1 (rTSST-1) was expressed and purified using an *Escherichia coli* expression system. The purity and molecular size of the recombinant toxins were determined by coomassie-blue-stained SDS-PAGE (Figure 1A). The concentration of endotoxins in the final purified rTSST-1 (0.406 mg·mL^−1^) was less than 0.001 EU·mL^−1^.

### 2.2. rTSST-1-Induced IL-1β and TNF-α Expression in Macrophages under the Action of ATP

**Figure 2:**
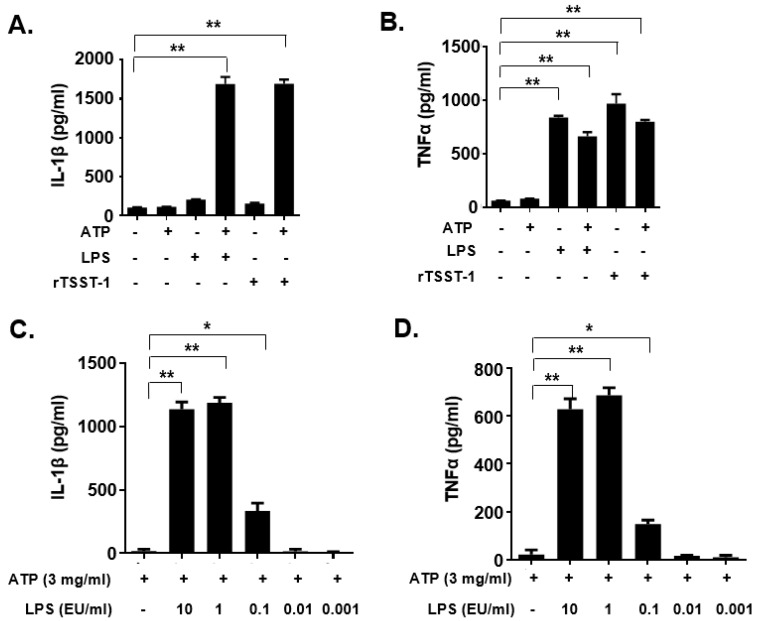
IL-1β and TNF-α expression in macrophages induced by rTSST-1 and LPS. The macrophages were stimulated with rTSST-1 or LPS, with or without the action of ATP. After stimulation, cell supernatants were collected and cytokines were determined by ELISA. The expression of IL-1β (**A**) and TNF-α (**B**) in LPS- or rTSST-1-stimulated macrophages. The expression of IL-1β (**C**) and TNF-α (**D**) in different concentrations of LPS-stimulated macrophages. The data are represented as mean ± SD of three independent experiments for each group (*n* = 3). * *p* < 0.05; ** *p* < 0.01.

To examine whether the rTSST-1 can induce inflammatory responses in mouse macrophages, rTSST-1 (0.01, 0.1 and 1 μg·mL^−1^) was used to stimulate cells under the action of ATP. After stimulation, mature IL-1β and TNF-α in the supernatants were detected by enzyme linked immunosorbent assay (ELISA). The results showed that both productions of IL-1β and TNF-α induced by rTSST-1 were dose-dependent under ATP-mediated activation of macrophages (Figure 1B,C). Notably, the secretion of IL-1β was not affected by TSST-1 priming only, but was clearly enhanced after ATP stimulation (Figure 2A,B). However, TNF-α production was highly induced by TSST-1 and it was not affected by ATP (Figure 2B). These results indicate that IL-1β expression is required by the ATP-mediated activation of macrophages. It has been reported that LPS and ATP can activate inflammasomes, leading to inflammatory cytokine expression. To confirm the effect of TSST-1-induced cytokine production not due to the contamination of LPS, we also analyzed the cytokine production induced by different concentrations of LPS and ATP. The results showed that a high concentration (higher than 0.1 EU·mL^−1^) of LPS induced IL-1β and TNF-α expression, but doses of LPS less than 0.01 EU·mL^−1^ did not induce the inflammatory cytokine expression in the cells (Figure 2C,D). The LPS concentration in our final dose of rTSST-1 was less than 10^−3^ EU·mL^−1^, indicating that the cytokine production induced by rTSST-1 is due to the activation of rTSST-1 itself, rather than the contamination of LPS.

### 2.3. rTSST-1-Induced IL-1β Secretion in Macrophages Is Dependent on TLR4 but Not on TLR2

**Figure 3:**
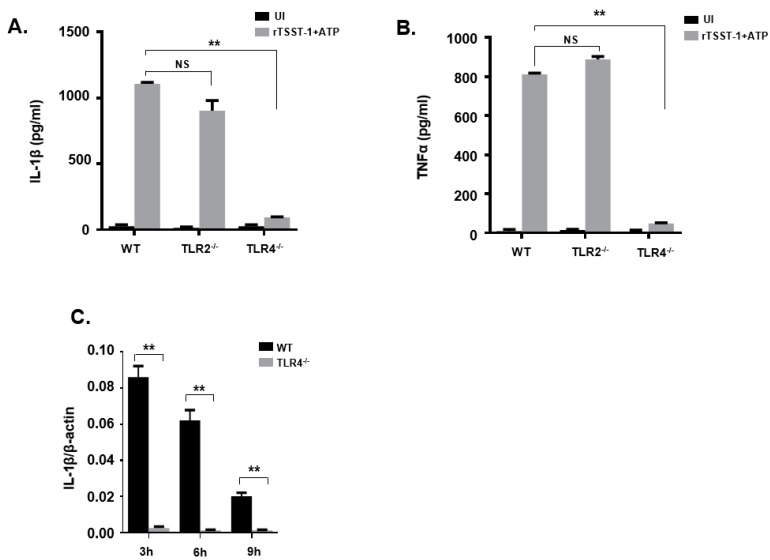
rTSST-1-induced inflammatory cytokine expression in macrophages from wild mice and gene knockout mice. Macrophages from C57BL/6 wild type (WT), TLR2^−/−^ and TLR4^−/−^ mice were unstimulated (UI) or stimulated with 1 ng·mL^−1^ rTSST-1 and 2 mM ATP. After indicated time stimulation, the expression of IL-1β (**A**) and TNF-α (**B**) in the cell supernatants were determined by ELISA and the mRNA expression of IL-1β (**C**) in the cells was determined by quantitative polymerase chain reaction (qPCR). The data are represented as mean ± SD of three independent experiments for each group (*n* = 3). ** *p* < 0.01.

TLR2 and TLR4 are cell surface receptors involved in the detection of different microbe-associated molecular patterns (MAMPs). The activation of TLR2 and TLR4 can activate cells, resulting in the production of inflammatory cytokines. To evaluate the role of TLR2 and TLR4 in rTSST-1+ATP-induced inflammatory response, peritoneal macrophages from wild type (WT), TLR2^−/−^ and TLR4^−/−^ mice were stimulated with rTSST-1+ATP, then inflammatory cytokines including IL-1β and TNF-α were detected by ELISA. After rTSST-1+ATP stimulation, both IL-1β and TNF-α secretion in macrophages of TLR4^−/−^ mice were markedly abolished while cytokine secretion was not affected in the macrophages of TLR2^−/−^ and WT mice (Figure 3A,B). These results demonstrate that TLR4 but not TLR2 was involved in the secretion of IL-1β and TNF-α induced by rTSST-1+ATP. To further determine the involvement of TLR4 in IL-1β secretion induced by rTSST-1+ATP in macrophages, we used rTSST-1+ATP to stimulate the peritoneal macrophages of WT mice and TLR4^−/−^ mice, respectively. After 3 h, 6 h, and 9 h stimulation, cell lysates were collected and RNA was extracted. A real-time fluorescent quantitative PCR test was performed. The result showed that IL-1β mRNA was not induced by rTSST-1+ATP in the macrophages of TLR4^−/−^ mice, indicating that TLR4 also affects the secretion of IL-1β at the transcription level (Figure 3C).

### 2.4. NLRP3 Inflammasome Was Involved in IL-1β Maturation and Secretion in rTSST-1-Stimulated Macrophages

**Figure 4:**
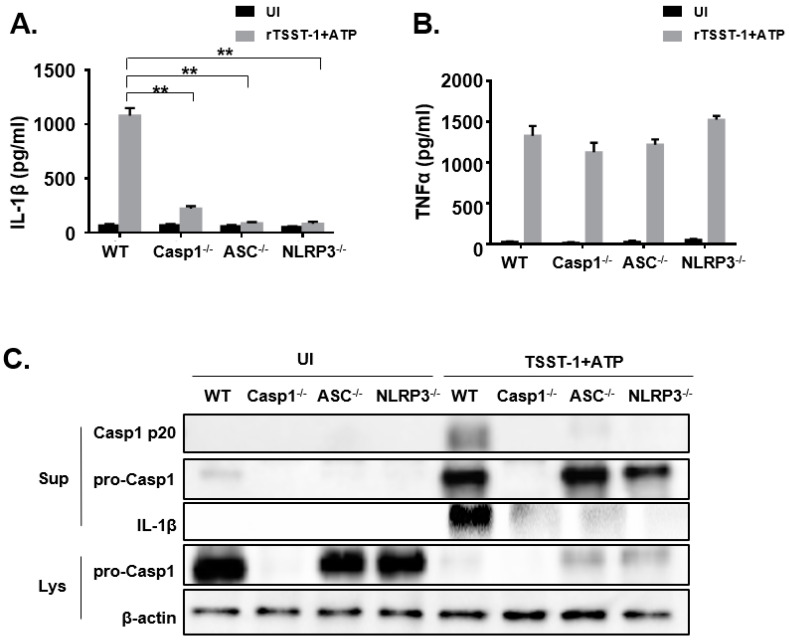
rTSST-1-induced IL-1β expression is dependent on the activation of NLRP3 inflammasome. Macrophages from WT, Casp1^−/−^, ASC^−/−^ and NLRP3^−/−^ mice were unstimulated (UI) or stimulated with 1 ng·mL^−1^ rTSST-1 and 2 mM ATP. After stimulation, expression of IL-1β (**A**) and TNF-α (**B**) in the supernatant were measured by ELISA. Caspase-1, pro-Caspase-1 and IL-1β in the cells were detected by Western blot (**C**). The data are represented as mean ± SD. of three independent experiments for each group (*n* = 3). ** *p* < 0.01.

To investigate the exact mechanism of rTSST-1-induced IL-1β secretion in macrophages, we hypothesized that rTSST-1 induced IL-1β production through inflammasome activation. Macrophages from WT, Caspase-1^−/−^, ASC^−/−^ and NLRP3^−/−^ mice were stimulated with rTSST-1+ATP, and the secretion of IL-1β and TNF-α in the cell supernatants were determined by ELISA (Figure 4A,B). Furthermore, cell lysates and supernatants were detected by Western blot. The ELISA results showed that IL-1β production was dramatically reduced in Caspase-1^−/−^, ASC^−/−^ and NLRP3^−/−^ macrophages (Figure 4A) but TNF-α expression was not affected (Figure 4B), indicating that rTSST-1-induced IL-1β secretion requires assembly of inflammasome proteins. Caspase-1 is a key factor in the maturation and secretion of IL-1β. The Western blot results showed that the mature Caspase-1 (Casp1 p20) was significantly expressed in the cell supernatant of WT mice macrophages, while there was no activated Caspase-1 expression detected in the cell supernatant of knockout mice, indicating that the activation of Caspase-1 depends on ASC and NLRP3. Similarly, the IL-1β expression of WT mice was significantly higher than in knockout mice. However, due to the activation of Caspase-1, a large number of Caspase-1 in the cell lysates was lysed into P20 fragments and secreted into the supernatants, resulting in less pro-Caspase-1 expression in the lysates of stimulated cells than those of the unstimulated cells (Figure 4C).

## 3. Discussion

TSST-1 is an important virulence factor of *S. aureus* strains. It can cause TSS, which is a serious infectious disease with rapid progression and high mortality. In order to understand the role of TSST-1 in host defense against *S. aureus* infection, we successfully obtained the high purity rTSST-1 toxin by using the *E. coli* prokaryotic expression system and investigated the effects of rTSST-1-induced inflammatory cytokine productions in the macrophages of mice. Our results showed for the first time that rTSST-1 can strongly induce inflammatory cytokine production in the macrophages of mice with participation of extrinsic ATP, and the inductive activities are dependent on the TLR4 and NLRP3 signaling pathway. It has been reported that multiple toxins from *S. aureus*, including α-Hemolysin (Hla), panton-valentine leukocidin (PVL) and leukocidin A/B (LukAB), were shown to activate the intracellular NLRP3 inflammasome in macrophages, monocytes and neutrophils, leading to pro-inflammatory cytokine release [32]. The well-known *staphylococcal* toxins that activate inflammasomes were pore-forming-toxins, so the activation of inflammasomes was thought to be limited to the pore-forming-toxins. In the present study, our results showed for the first time that TSST-1, a non-pore-forming toxin of *S. aureus*, can activate inflammasomes and induce strong inflammatory responses in macrophages in the participation of extrinsic ATP.

TSST-1 is a typical bacterial superantigenic toxin that can directly bind to the major histocompatibility complex class II molecules on macrophages or dendritic cells and T cell receptors [26,27]. This subsequently leads to a large proliferation of T cells and high production of proinflammatory cytokines including interleukin (IL)-2 and interferon (IFN)-γ [26,27] which causes life-threatening TSS. Narita et al. reported that vaccination with a non-superantigenic mutant TSST-1 (mTSST-1) protected mice against *S. aureus* infection dependent on increased number of IL-17A-produced cells [33]. They found that rTSST-1 stimulation induced the expression of IFN-γ and IL-17 in spleen cells from mTSST-1-vaccinated mice, and the expression of cytokines was dependent on its superantigenic activity. Cui et al. reported that immunization with glutathione S-transferase (GST)-mTSST-1 induced TSST-1-neutralizing antibody production, and combination of TSST-1 with the antibody from GST-mTSST-1-immunized mice neutralized the superantigenic activity of TSST-1, resulting in the reduction of IFN-γ and TNF-α production in spleen cells of mice [34]. These results indicated that TSST-1 could induce adaptive immune responses, neutralizing antibody production. In this experiment, rTSST-1 could directly induce macrophages to produce IL-1β and TNF-α under the action of ATP, suggesting that superantigenic activity did not mediate TSST-1-induced inflammatory cytokine production. These studies indicate that TSST-1 can be developed as a novel molecular target for future adjuvant therapy to treat *S. aureus* infections.

Some TLRs are on the surface of cell membrane and detect different microbe-associated molecular patterns (MAMPs). After the activation of TLR induced by microbial molecules, interleukin-1 receptor-related kinases and TNF receptor-related factors are activated through the MyD88 pathway, leading to the expression of pro-IL-1β, pro-IL-18 and NLRP3 at the transcriptional level through the activation of NF-κB signaling pathway. In our study, we observed that the secretion of IL-1β was significantly decreased in TLR4^−/−^ macrophages stimulated with TSST-1+ATP, but it was not affected in TLR2^−/−^ macrophages, indicating that TLR4 instead of TLR2 is necessary for IL-1β secretion.

However, unlike other toxins of *S. aureus*, such as PVL, which directly induces the release of IL-1β [30], only TSST-1 did not induce the release of IL-1β in this study, which indicates that TSST-1 is different from the pore-forming-toxins of *S. aureus*. In order to secrete IL-1β, TSST-1 as primary stimuli induced the synthesis of pro-IL-1β and provided signals for secondary stimuli ATP to enhance immune response. It has been reported that secondary stimuli can induce cell necrosis with the activation of caspase-1, leading to IL-1β secretion [35]. Our study indicates that only TSST-1 did not induce cell necrosis and provide the important signal for the production of IL-1β. 

In conclusion, our study investigated the TSST-1-induced maturation and secretion of IL-1βvia the TLR4/NLRP3 pathway in macrophages. Our results showed that in response to TSST-1+ATP stimulation, the NLRP3 inflammasome is assembled and caspase-1 is activated in macrophages in vitro, indicating that the NLRP3 inflammasome is critically involved in this process. Even though our study is limited to cell level, the knockout mice used in this study provide a useful tool for investigating the role of inflammasomes in host defense against microbial infection. The role of the NLRP3 inflammasome in TSST-1-host interactions may need to be studied in vivo using inflammasome component knockout mice in the future. Our study was the first to show a pathophysiological link between staphylococcal superantigenic toxins and the NLRP3 inflammasome, providing a new therapeutic strategy for TSST-1-induced inflammatory diseases.

## 4. Materials and Methods

### 4.1. Toxins

The recombinant plasmid with the *tst-1* gene, pGEX-TSST-1 was constructed and prepared as the methods described previously [34]. The pGEX-TSST-1 was transformed into *Escherichia coli* BL21. After overnight culture, the bacterial cells were diluted 1:100 in 250 mL of LB (LAND BRIDGE, Beijing, China) medium containing 100 μg·mL^−1^ ampicillin (Solarbio, Beijing, China). *E. coli* cells were grown until optical density was reached (0.5–0.8 at 600 nm). Isopropyl-1-thio-b-d-galactopyranoside (0.2 mmol·L^−1^) at 16 °C was added to induce the expression of recombinant proteins. After overnight culture, bacterial pellets were collected and resuspended in sterilized PBS. GST-rTSST-1 was purified using the Glutathione Sepharose 4B (GE Healthcare Life Sciences, Marlborough, MA, USA) as previous studies described [1,34]. After endotoxin removal, the concentration of LPS in the purified proteins was less than 10^−3^ EU·mL^−1^. Eventually, the purified proteins were determined by Bradford assay (Bio-Rad, Hercules, CA, USA) and analyzed by SDS-PAGE.

### 4.2. Macrophages

Wild type C57BL/6 mice, TLR2^−/−^ mice and TLR4^−/−^ mice with the C57BL/6 background were purchased from the Chongqing Academy of Chinese Materia Medical (Chongqing, China). Caspase-1^−/−^, ASC^−/−^, and NLRP3^−/−^ mice with the C57BL/6 background were kindly provided by Feng Shao from the National Institute of Biological Sciences, Beijing, China. All mice were maintained under specific-pathogen-free conditions and used at 8–10 weeks old. The mice were intraperitoneally injected with 2 mL 4% thioglycollate medium (Eiken Chemical, Tokyo, Japan), then 3–4 days later, peritoneal exudate cells (PECs) were collected by peritoneal lavage as described before [9]. Cell culture prior to stimulation was described in our previous study [22]. All of the animal experiments were approved by the Ethics Committee of Southwest University and carried out in accordance with the laboratory animal care principles of the National Institutes of Health, China.

### 4.3. Enzyme Linked Immunosorbent Assay (ELISA)

The macrophages were stimulated with rTSST-1 at a concentration of 0.01 μg·mL^−1^, 0.1 μg·mL^−1^, 1 μg·mL^−1^ and cultured at 37 °C in a humidified 5% CO_2_ environment for 3 h. Then, ATP (2 Mm; Roche Diagnostics GmbH, Mannheim, Germany) was added to the cells, which were then incubated for an additional 18 h. After incubation, the cell-free culture supernatants were collected to measure the concentration of cytokines (IL-1β and TNF-α) using ELISA kits (eBioscience, San Diego, CA, USA).

### 4.4. Real-Time Fluorescent Quantitative PCR

The macrophages were stimulated with rTSST-1 (1 μg·mL^−1^) + ATP (2 mM) for 3, 6 and 9 h. After stimulation, total RNA was extracted using the RNA pre-pure Kit (TIANGEN, Beijing, China) according to the manufacturer’s instructions. Then RNase-free DNase (TIANGEN) was added to RNA (0.2 µg) to remove the DNA, after which the 250R iScript advanced cDNA Synthesis kit (Bio-rad) was used to obtain cDNA. Quantitative real-time RT-PCR was performed on a Bio-rad CFX 96 (Hercules, CA, USA) using an soFast Eva Green Super-Mix (Bio-rad) according to the manufacturer’s instructions. The primers for quantitative real-time RT-PCR were as follows: IL-1β, 5′-GAAATGCCACCTTTTGACAGTG-3′ (forward) and 5′-TGGATGCTCTCATCAGGACAG-3′ (reverse); β-actin, 5′-TGGAATCCTGTGGCATCCATGAAAC-3′ (forward) and 5′-TAAAACGCAGCTCAGTAACAGTCCG-3′ (reverse). The relative expression was analyzed against the expression level of β-actin.

### 4.5. Western Blot Analysis

The cells were cultured on 12-well plates (1 × 10^6^ cells/well) in RPMI 1640 with 10% FCS for 2 h. After washing, the cells were stimulated with TSST-1 in Opti-MEM (Invitrogen) as described above. Cell supernatants were collected after 24 h stimulation and the cells were lysed using a radioimmune precipitation assay (RIPA) buffer (Beyotime, Beijing, China). The protein concentrations were measured using a BCA Protein Assay Kit (Beyotime, Beijing, China) and 12–15% SDS-PAGE gel was used to analyze the proteins in the samples, which were then transferred to a polyvinylidene difluoride (PVDF) membrane to detect the target protein. After this, the membranes were blocked with 5% nonfat milk and immunoblotted with the appropriate antibody, and protein bands were detected using the ECL detection reagent (Beyotime, Beijing, China). Different antibodies were used in this study including Biotinylated anti-mouse IL-1β (AdipoGen, San Diego, CA, USA), Anti-Caspase-1 (p20) (AdipoGen), Anti-Caspase-1 (p45) (AdipoGen), HRP-conjugated mouse anti-goat or anti-mouse IgG (Beijing, China). Anti-actin was used as a loading control for the cell lysates.

### 4.6. Statistical Analysis

The statistical analysis was performed using GraphPad Prism software and the data were represented as mean ± SD of three independent experiments for each group (*n* = 3). Student’s *t*-test and ANOVA were used to analyze the significant differences between the two groups or three groups, respectively. Statistical significance was defined as *p* value (* *p* < 0.05 and ** *p* < 0.01).

## Data Availability

All the data generated for this study are included in the article.
